# Inhibition of autophagy‐dependent pyroptosis attenuates cerebral ischaemia/reperfusion injury

**DOI:** 10.1111/jcmm.16483

**Published:** 2021-05-02

**Authors:** Hui Liu, Zongbo Zhao, Tao Wu, Qiu Zhang, Fenying Lu, Jie Gu, Tingwang Jiang, Jianzhong Xue

**Affiliations:** ^1^ Department of Neurology and Institute of Neurology The Affiliated Changshu Hospital of Xuzhou Medical School Suzhou Jiangsu China; ^2^ Department of Gastroenterology The Affiliated Changshu Hospital of Xuzhou Medical School Suzhou Jiangsu China; ^3^ Department of Key Laboratory The Affiliated Changshu Hospital of Xuzhou Medical School Suzhou Jiangsu China; ^4^ Jiangsu Key Laboratory of Brain Disease Bioinformation Xuzhou Medical University Xuzhou Jiangsu China

**Keywords:** autophagy, cerebral ischaemia/reperfusion injury, inflammation, pyroptosis, Spautin‐1

## Abstract

Autophagy is closely associated with cerebral ischaemia/reperfusion injury, but the underlying mechanisms are unknown. We investigated whether Spautin‐1 ameliorates cerebral ischaemia/reperfusion injury by inhibiting autophagy and whether its derived pyroptosis is involved in this process. We explored the mechanism of Spautin‐1 in cerebral ischaemia/reperfusion. To answer these questions, healthy male Sprague‐Dawley rats were exposed to middle cerebral artery occlusion for 60 minutes followed by reperfusion for 24 hours. We found that cerebral ischaemia/reperfusion increased the expression levels of autophagy and pyroptosis‐related proteins. Treatment with Spautin‐1 reduced the infarct size and water content and restored some neurological functions. In vitro experiments were performed using oxygen‐glucose deprivation/reoxygenation to model PC12 cells. The results showed that PC12 cells showed a significant decrease in cell viability and a significant increase in ROS and autophagy levels. Spautin‐1 treatment reduced autophagy and ROS accumulation and attenuated NLRP3 inflammasome‐dependent pyroptosis. However, these beneficial effects were greatly blocked by USP13 overexpression, which significantly counteracted the inhibition of autophagy and NLRP3 inflammasome‐dependent ferroptosis by Spautin‐1. Together, these results suggest that Spautin‐1 may ameliorate cerebral ischaemia‐reperfusion injury via the autophagy/pyroptosis pathway. Thus, inhibition of autophagy may be considered as a promising therapeutic approach for cerebral ischaemia‐reperfusion injury.

## 1 INTRODUCTION

Stroke is one of the leading causes of death and disability worldwide, and more than 80% of stroke cases are ischaemic stroke.^1^ Reperfusion of the ischaemic area through drugs or early thrombolysis to restore the supply of oxygen and glucose is the main clinical strategy in ischaemic stroke.^2^ Reperfusion can restore blood supply, reduce infarct size, and tissue dysfunction caused by ischaemia,^3^ but it can also cause the accumulation of reactive oxygen species (ROS) and subsequent oxidative stress, which can exacerbate ischaemic brain injury.^4^ Cerebral ischaemia/reperfusion injury (CI/RI) has become an increasingly serious challenge for stroke patients. Studies have shown that inflammatory response, oxidative stress, autophagy and apoptosis are the main cause of CI/RI.^5^, ^6^, ^7^ Currently, the management of CI/RI remains a thorny issue that needs to be addressed.

Autophagy is an intracellular degradation process essential for cellular homoeostasis that degrades damaged or aged organelles and proteins through autolysosomes. Autophagy has been reported to be common in ischaemic stroke,^8^, ^9^ and excessive autophagy has negative effects on cerebral ischaemia‐reperfusion.^10^ In the process of autophagy activation, microtubule‐associated protein 1 light chain 3‐I (LC3‐I) can be hydrolysed to LC3‐II, and therefore, the ratio of LC3‐II/LC3‐I and Beclin1 is widely used as a marker of autophagy activation levels.^11^ Oxidative stress and inflammation are important pathological processes of CI/RI.^12^, ^13^ In middle cerebral artery occlusion/reperfusion (MCAO/R) rat model, the content of inflammatory cytokines was increased, accompanied by reactive oxygen species (ROS) accumulation.^14^, ^15^ Therefore, inhibiting the production of inflammatory cytokines and reducing the accumulation of ROS may be crucial for the prognosis and treatment of cerebral ischaemia‐reperfusion.

Vps34 consists mainly of the N‐terminal C2 structural domain, the CCD and the C‐terminal phospholipid kinase structural domain, and the C2 structural domain is able to bind Beclin1, forming the Vps34‐Beclin1 complex. Spautin‐1 is an inhibitor of USP10 and USP13. USP10 and USP13, two ubiquitin‐specific peptidases, can target the Beclin1 subunit of Vps34‐Beclin1 and promote the degradation of the Vps34 complex.^16^ USP13 is a deubiquitinase that has been shown to modulate the stability of tumour‐related proteins and inflammatory responses.^16^, ^17^

The name of this study was to investigate whether Spautin‐1 is involved in the inflammatory response during CI/RI and its underlying molecular mechanism using an in vivo rat model of MCAO/R and in vitro PC12 cell model of oxygen‐glucose deprivation/reoxygenation (OGD/R). Subsequently, the effect of Spautin‐1 on CI/RI was further investigated.

## 2 MATERIALS AND METHODS

### 2.1 Cell culture and the OGD/R model

PC12 cells (Cell Bank of Chinese Academy of Sciences) were cultured in DMEM (Gibco) medium supplemented with 10% foetal bovine serum (FBS, Wisent, Canada) and dual antibiotics (100 µg/mL of penicillin and 100 µg/mL of streptomycin, Beyotime, China). Cells were cultured in an ordinary incubator (5% CO_2_ and 21% O_2_) at 37°C.

The OGD/R modelling method of PC12 cells was referred to previous research.^18^ In brief, PC12 cells were hypoxic for 12 hours at 37°C using DMEM without glucose and FBS in a hypoxic chamber filled with 1% O_2_, 5% CO_2_, and 94% N_2_, and then re‐oxygenated DMEM medium containing 10% FBS for 4 hours. Spautin‐1 (HY‐12990, MCE, China) was dissolved in DMSO. PC12 cells were divided into three groups: (1) Control group: cells were cultured under normal conditions; (2) OGD/R group: cells were subjected to 24 hours of hypoxia and hypoglycaemia followed by 4 hours of reoxygenation and re‐glycemation; and (3) OGD/R + Spautin‐1 group: cells were pre‐treated with 10 μmol/L Spautin‐1 for 24 hours and followed by OGD/R modelling.

### 2.2 Cell viability assay

The PC12 cells were seeded at a density of 1 × 10^4^ cells/well and incubated overnight. Spautin‐1 solution at the concentrations of 0, 5, 10 and 20 μmol/L was then added to the wells and incubated for 24 hours. In short, cells were treated with 0.25 μg/μL MTT for 4 hours, and formazan was dissolved by DMSO‐SDS lysis solution. The absorbance of the supernatant was read at 570 nm, and the measurements were reported as the percentage of control.^19^ The viability was calculated by Equation (1) as follows:

(1) $$\text{Viability}\;\left(\%\right)=\left(1‐\frac{\text{mean}\;\text{OD}\;\text{of}\;\text{sample}}{\text{mean}\;\text{OD}\;\text{of}\;\text{control}}\right)\times 100\%$$

### 2.3 TEM observation

After the PC12 cells were treated according to the conditions of each group, the intrinsic culture medium was discarded, and the culture medium containing PBS was used to wash the cells for three times. Cells were collected and immediately soaked in 2.5% buffered glutaraldehyde at 4°C for 2 hours, dehydrated with acetone gradient 2 times (30%, 50%, 75%, 80%, 95% and 100%), then embedded in Pon‐812 (90529‐77‐4, SPI, USA), and polymerized at 60°C for 48 hours. Ultrathin sections were observed under the TEM (HT7800; Hitachi) after electron staining with 1% uranyl acetate and lead citrate.

### 2.4 Western blot

Western blot analysis was designed to assess the expression of autophagy and pyroptoysis‐related proteins. Target protein concentrations were determined using a BCA protein assay kit (Pierce). Equal amounts of proteins were separated by 10% sodium dodecyl sulphate‐polyacrylamide gel electrophoresis and transferred to polyvinylidene fluoride membranes. The membranes were than immersed in Tris‐buffered saline Tween (TBST) containing skimmed milk and left for 1 hour at room temperature. Thereafter, the membrane was added with a suitable concentration of primary antibody NLRP3 (ab214185, Abcam), USP13 (ab99421, Abcam), NF‐κB (abs131170, absin), Beclin 1 (3738, CST), GSDMD (93709s, CST), GSDMD‐N (ab215203, Abcam), Cleaved IL‐1β (AF4006, Affinity), ASC (67824, CST), LC3 I and LC3 II (12741, CST) and GAPDH (AF7021, Affinity) at 4°C, and then washed three times with TBST. After that, the membrane was incubated with the horseradish peroxidase‐labelled secondary antibody Goat Anti‐Mouse IgG (H + L) HRP (1:5000, No. S0002, Affinity) or Goat Anti‐Rabbit IgG (H + L) HRP (1:5000, No. S0001, Affinity) at room temperature for 2 hours. Afterwards, the blot was detected using enhanced chemiluminescence. The gel analysis system scanned each strip protein, and the grey value of the strip was measured by image analysis software (Image J).

### 2.5 Immunofluorescence (IF)assay

The levels of USP13 and NLRP3 in brain tissues and SV‐HUC‐1 cells were assessed by immunofluorescence. Brain tissues were removed, fixed in 4% paraformaldehyde solution for 24 hours at 4°C. After embedding in paraffin, the tissues were cut into 4‐µm‐thick continuous coronal brain slices. Brain tissues and cells were blocked with 5% BSA blocking solution for 60 minutes at room temperature, followed by washing with PBS. Samples were then incubated with USP13 antibody or NLRP3 antibody at 4°C overnight. Sections were washed 3 times (5 minutes each time) with ice‐cold PBS and further incubated with goat anti‐rabbit IgG‐AF488 (Affinity, USA) secondary antibody.

Cells were fixed with 4% paraformaldehyde for 20 minutes and permeabilized with 0.5% Triton X‐100 for 20 minutes. Cells were blocked for 60 minutes at room temperature using 5% BSA blocking solution and then washed with PBS. Thereafter, cells were incubated with 1:200 dilution of primary antibodies (rabbit anti‐NLRP3 antibody, Affinity, USA) for overnight at 4°C. Following overnight incubation, cells were washed three times with PBS and incubated for 2 hours in dark with second antibody (goat anti‐rabbit, Affinity).

Later, the nuclei of cells were stained with 4′,6‐diamidino‐2‐phenylindole (DAPI, C0060, Solarbio); representative fluorescence images were obtained using fluorescence microscope (BX63, Olympus).

### 2.6 The detection of ROS

For the detection of ROS in cells, cells were subjected to a ROS assay using the DCFA‐DA reactive oxygen ROS fluorescent probe (D6470, Solarbio) according to the manufacturer's instructions. Experimental cells were washed twice with PBS and then incubated in DMEM medium containing 10% FBS and DCFH‐DA dye for 30 minutes and analysed using a flow cytometer (BD FACS Calibur Flow Cytometer) to measure the intensity of DCFH‐DA fluorescence.

### 2.7 Lentiviral overexpression of USP13 and administration

To further investigate the effect of Spautin‐1 on USP13, we overexpressed USP13 in PC12 cells. The recombinant lentivirus vectors for USP13 and empty vector were provided by Genechem. PC12 cells in a 6‐well plate at a concentration of 5 × 10^4^ cells/wells were incubated with 25 × HiTransG lentivirus infection reagent (Genechem Co., Ltd.) and 1 × 10^7^ lentivirus with empty vectors/USP13 overexpression for 12 hours, and then, the normal culture medium was replaced. PC12 cells were specifically divided into five groups: Control group (cell with empty vector), Model group (OGD/R, cell with empty vector), Model + USP13‐OE group (OGD/R, cell with USP13 overexpression), Model + Spautin‐1 group (OGD/R, cell with empty vector was treated with 10 μmol/L Spautin‐1), and Model + USP13‐OE + Spautin‐1 group (OGD/R, cell with USP13 overexpression was treated with 10 μmol/L Spautin‐1).

### 2.8 Animals and cerebral ischaemia‐reperfusion model

Adult male SD rats (200‐220 g) were purchased from SPF (Beijing) Biotechnology Co. Ltd. (production licence: SCXK (Beijing) 2019‐0010). They were housed at a temperature of 22 ± 1°C with a 12 hours light/dark cycle. The procedures were carried out in accordance to the Guidelines for Laboratory Animal Care. The animal experimental protocol was approved by the Animal Care and Use Committee of Affiliated Changshu Hospital of Xuzhou Medical School. This rat cerebral ischaemia model was made from middle cerebral artery occlusion.^20^ The animals were arbitrarily isolated into four groups and 15 rats each: (1) Sham group, rats without carotid occlusion; (2) I/R group, carotid artery occlusion was performed for 60 minutes followed by reperfusion for 24 hours; and (3) I/R + Spa group, pre‐treatment with Spautin‐1 (20 mg/kg/d) before MCAO. In the I/R + Spa group, rats were injected with Spautin‐1 intraperitoneally twice a day for 3 days, and the model was made 1 hour after the last administration on the third day. In the Sham group and I/R group, rats were injected with saline intraperitoneally. The animals were anesthetized with injection of 10% chloral hydrate, and both common carotid arteries were separated from the vagus nerves. The common carotid arteries were clipped by a vascular clamp for 30 minutes and reperfused. The blood flow restoration was confirmed in the carotid arteries via observation. The rats in sham group underwent all surgical procedures except that the arteries were not ligated.

### 2.9 Neurological test

According to the observation of different degrees of paralysis in rats, the paralysis is divided into 5 levels: score 0, no neurological deficit; score 1, adduction and flexion of the contralateral forelimb during tail lifting (mild neurological deficit); score 2, circling during walking (moderate neurological deficit); score 3, rolling to its side during walking due to hemiplegia (severe neurological deficit); and score 4, no voluntary movements and impaired consciousness.^21^ Only rats with a score of 1 to 2 were used in this study.

### 2.10 Determination of water content and leakage of Evans Blue in brain tissue

Rats were decapitated rapidly after euthanasia, brain tissue was removed on ice, and after removing the meninges, lower brainstem and cerebellum, the remaining brain tissue was weighed (wet weight). Then, the brain tissue was baked at 105℃ until the difference between the two weights measured was <0.2 mg. The water content was calculated by Equation (2) as follow:

(2) $$\text{Brain tissue}\;\text{water content}\;\left(\%\right)=\frac{\left(\text{wet}\;\text{weight}‐\text{dry}\;\text{weight}\right)}{\text{wet}\;\;\text{weight}}\times 100\%$$

During the disruption of the blood‐brain barrier (BBB) after I/R, EB readily penetrates the BBB and stains brain tissue blue. Therefore, we used EB to investigate whether Spautin‐1 pre‐treatment could inhibit the disruption of the BBB. 4% Evans blue dye dissolved in 0.9% saline was injected into the tail vein of each group of rats. Two hours after injection, the rats were anesthetized and the hearts were perfused with saline to remove the Evans blue dye. The ischaemic hemispheres were immediately collected, homogenized with 50% trichloroacetic acid, and centrifuged at 11 000 *g* for 5 minutes at 4°C. The supernatant was collected and determined its optical density (OD) at 620 nm. Finally, the EB content was calculated from the standard curve of EB.

### 2.11 Pathological examination of brain tissue

Immediately after reperfusion, brain tissues were removed and frozen at −20°C for 20 minutes. The brain tissues were then cut into 2‐mm‐thick transverse slices, and the slices were immersed in 2% solution 2,3,5‐Triphenyltetrazolium chloride (TTC, Sigma) for 10 minutes at 37°C. Representative images were taken by the camera, and the ratio of the infarct area (white area) was calculated as Equation (3).

(3) $$\text{infarct}\;\text{rate}=\frac{\text{infarct}\;\text{area}}{\text{total}\;\text{area}}\times 100\%$$

Rat brains were collected and fixed with 4% paraformaldehyde at room temperature for 24 hours. The brain tissues were embedded in paraffin and cut into 4 µm sections, dewaxed and rehydrated, and then stained with haematoxylin and eosin (H&E) as described in a previous research.^22^ Sections were observed and photographed under an optical microscope (Olympus, Japan).

### 2.12 Statistical analysis

Statistical analysis was performed with GraphPad Prism 8.0. Data are presented as the mean ± standard deviation (SD). Comparison among multiple groups was analysed with one‐way analysis of variance (ANOVA), after which pairwise comparison was analysed by Tukey's post hoc test. Values of *P <* .05 were considered to be significant.

## 3 RESULTS

### 3.1 Spautin‐1 pre‐treatment improves cell viability and reduces ROS accumulation and autophagic microsomal number in OGD/R‐treated PC12 cells

**FIGURE 1:**
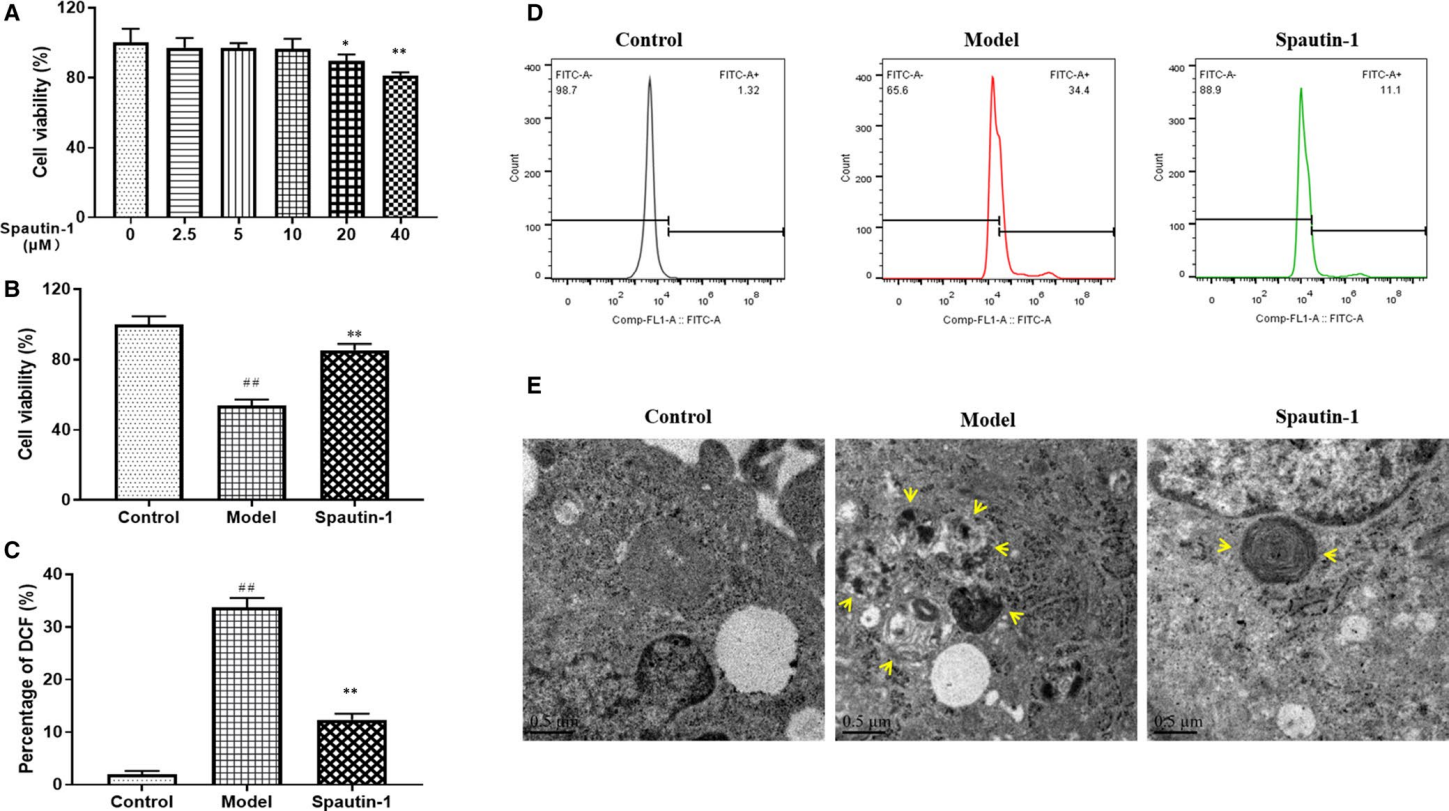
Spautin‐1 pre‐treatment increases cell viability and reduces ROS accumulation and autophagic microsome number in OGD/R‐treated PC12 cells. A, Concentration screening of the inhibitor Spautin‐1; B, Cell viability of different groups; C, The percentage of ROS in OGD/R modelling cells; D, Flow cytometry analysis of DCFH‐DA dye treatment of different groups of cells; E, Cell autophagosome electron micrographs. Control, the control group without OGD/R; Model, the model group with OGD/R; Spautin‐1, the group with OGD/R plus Spautin‐1. Data are expressed as the mean ± SD, ^#^ *P* < .05 and *^##^P* < .01 compared with the Control group; *P < .05 and **P < .01 compared with the Model group

We generated an in vitro model of OGD/R induced cerebral ischaemia‐reperfusion in PC12 cells. First, PC12 cells were cultured to test the effect of Spautin‐1 on cell viability, and an appropriate concentration was selected for administration (Figure 1A). Based on the results of the cell viability experiments, 10 μmol/L was selected as the concentration for the administration of Spautin‐1, and it was found that Spautin‐1 administration significantly increased cell viability (Figure 1B). Oxidative stress is one of the important mechanisms in the pathology of cerebral ischaemia‐reperfusion injury.^12^ The ROS content was significantly increased in the model group compared with the control group, but decreased with the addition of Spautin‐1 (Figure 1C,D). As shown in Figure 1E, cells in the control group had normal structure with intact nuclei and uniform chromatin distribution; the model group cells had uneven chromatin distribution, some organelles were dissolved, and the structure was unclear; the cells in the Spautin‐1 group showed improvement compared with the model group; and the number of autophagic microsomes was significantly reduced. These results suggested that Spautin‐1 could improve cell viability and reduces the ROS accumulation and the number of autophagic microsomes in an in vitro ischaemia‐reperfusion model.

### 3.2 Spautin‐1 ameliorates the unfavourable milieu by attenuating the expression of autophagy and pyroptosis‐related proteins in OGD/R‐treated PC12 cells

**FIGURE 2:**
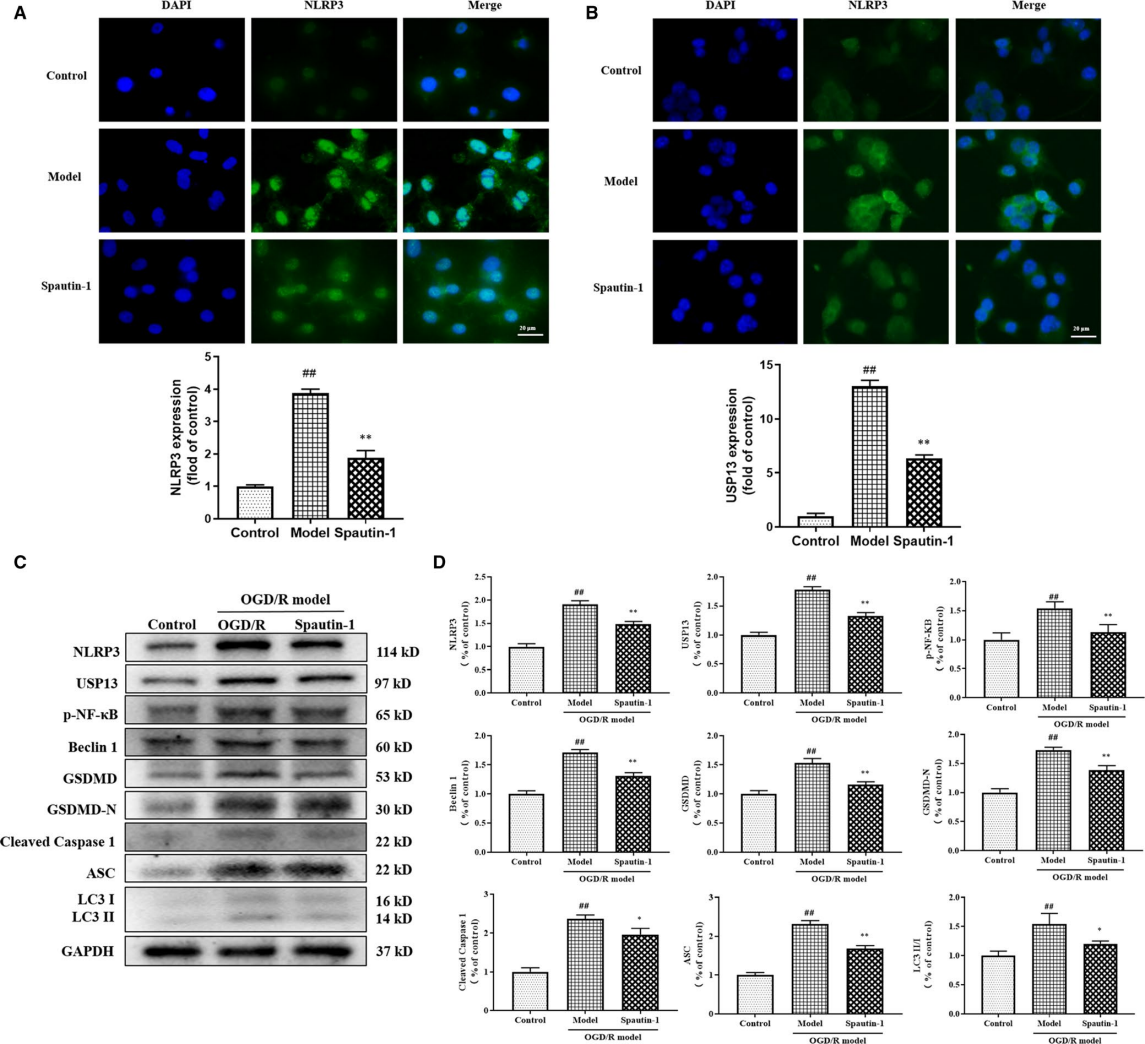
Spautin‐1 improves the unfavourable milieu by attenuating the expression of autophagy and pyroptosis‐related proteins in OGD/R‐treated PC12 cells. A, The expression of NLRP3 in the OGD/R cell by immunofluorescence; B, The expression of USP13 in the OGD/R cell by immunofluorescence; C, The protein levels of NLRP3, USP13, p‐NF‐κB, Beclin 1, GSDMD, GSDMD‐N, Cleaved Caspase‐1, ASC and LC3 II/LC3 I by Western blot; D, Densitometric analysis of autophagy and pyroptosis‐related proteins relative expression. Control, the control group without OGD/R; Model, the model group with OGD/R; Spautin‐1, the group with OGD/R plus Spautin‐1. Data are expressed as the mean ± SD, ^#^ *P* < .05 and *^##^P* < .01 compared with the Control group; *P < .05 and **P < .01 compared with the Model group

Next, the expression of NLRP3 and USP13 in PC12 cells was detected by immunofluorescence staining, and statistical analysis showed that the highest expression of NLRP3 and USP13 was found in the model group, and the fluorescence intensity of both NLRP3 and USP13 in the Spautin‐1‐treated group was lower than that in the model group, but higher than that in the control group (Figure 2A,B). Western blot was used to detect expressions of autophagy and pyroptosis‐related proteins. In OGD/R model group, the expressions of NLRP3, USP13, p‐NF‐κB, Beclin1, GSDMD, GSDMD‐N, Cleaved Caspase‐1, ASC and LCII/I were significantly increased. However, this upward trend was suppressed in the Spautin‐1 group (*P *< .01, Figure 2C,D).

### 3.3 Spautin‐1 regulates expressions of NLRP3 and Beclin1 by suppressing USP13 in OGD/R‐treated PC12 cells

**FIGURE 3:**
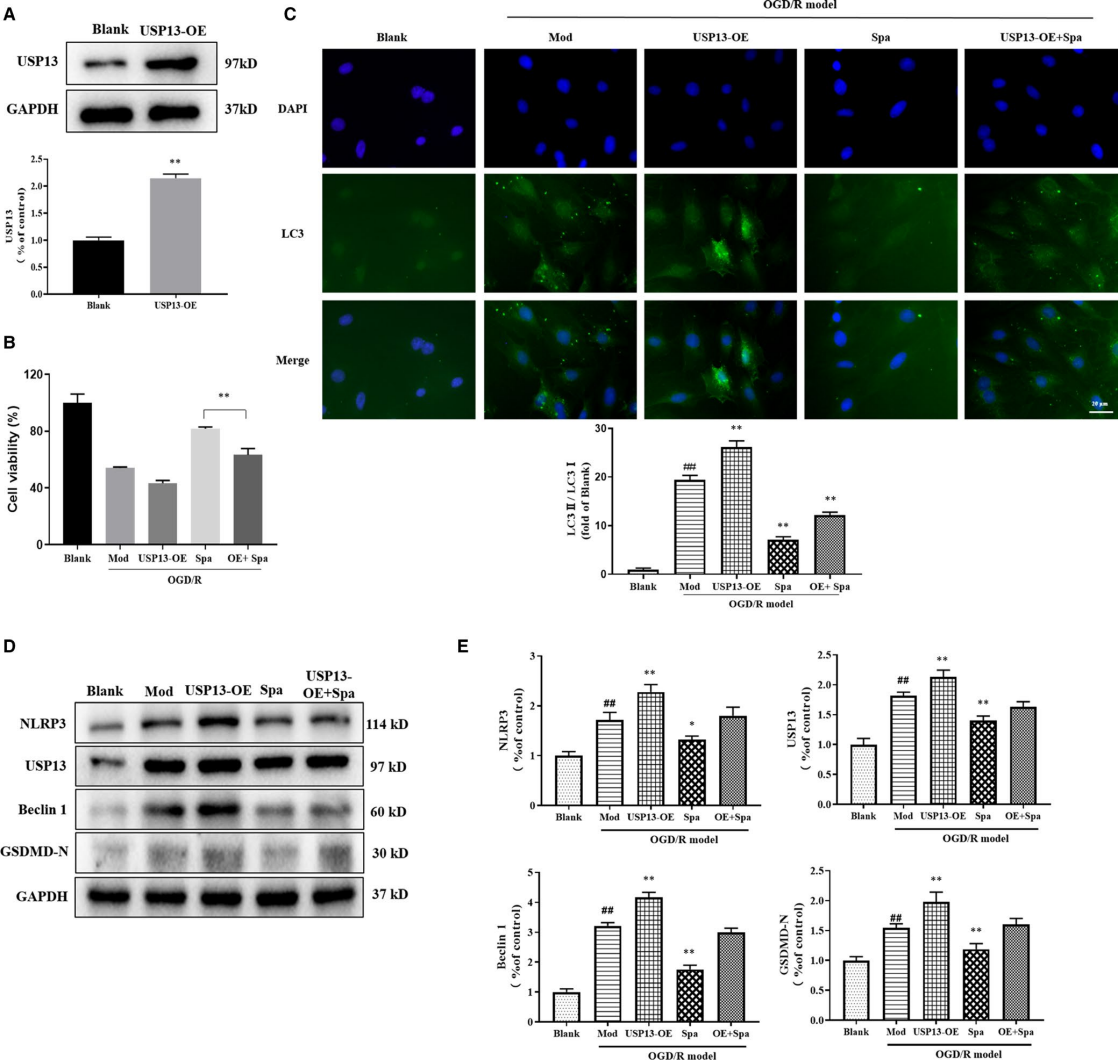
Spautin‐1 regulates NLRP3 and Beclin1 expression by suppressing USP13 in OGD/R‐treated PC12 cells. A, Western blot analyses of USP13 from PC12 cells transfected with blank carrier or USP13; B, Cell viability of different groups; C, Representative images of GFP‐LC3 and DAPI; D, The protein levels of NLRP3, USP13, Beclin 1 and GSDMD‐N by Western blot; E, Densitometric analysis of NLRP3, USP13, Beclin 1 and GSDMD‐N relative expression. Blank, the blank carrier group; Mod, the model group with OGD/R; USP13‐OE, the cell overexpressed USP13 group with OGD/R; Spa, the OGD/R model group with Spautin‐1; OE + Spa, the cell overexpressed USP13 group with OGD/R plus Spautin‐1. Data are expressed as the mean ± SD, ^#^ *P* < .05 and *^##^P* < .01 compared with the Control group; *P < .05 and **P < .01 compared with the Model group

We overexpressed USP13 in PC12 cells to further validate the effect of Spautin‐1 on ischaemia‐reperfusion cell model. As shown in Figure 3A, the expression of USP13 in the USP13‐OE group was obviously higher than that in the Blank group (*P* < .01), indicating the success of the USP13 overexpression construct. The cell viability of Spa group was higher than that of the OE + Spa group (Figure 3B). As the conversion of LC3‐I to LC3‐II is a classic marker for autophagy formation,^23^ the effect of Spautin‐1 on autophagy was then detected using fluorescence of LC3 in cells overexpressing USP13. Compared with the control group, a significant fluorescent signal was detected in the OGD/R‐treated group, but LC3 expression was significantly lower in the Spa group than in the Mod group. Meanwhile, LC3 expression was increased in the OE + Spa group compared with the Spa group (Figure 3C). Overexpression of USP13 in PC12 cells almost reversed the Spautin‐1‐mediated decrease in NLRP3, USP13, Beclin1 and GSDMD‐N expression levels (Figure 3D,E). These results suggested that the effects of Spautin‐1 on the expressions of autophagy and pyroptosis‐related proteins were dependent on the USP13 abundance.

### 3.4 Spautin‐1 pre‐treatment ameliorates the injury of cerebral I/R in rats

**FIGURE 4:**
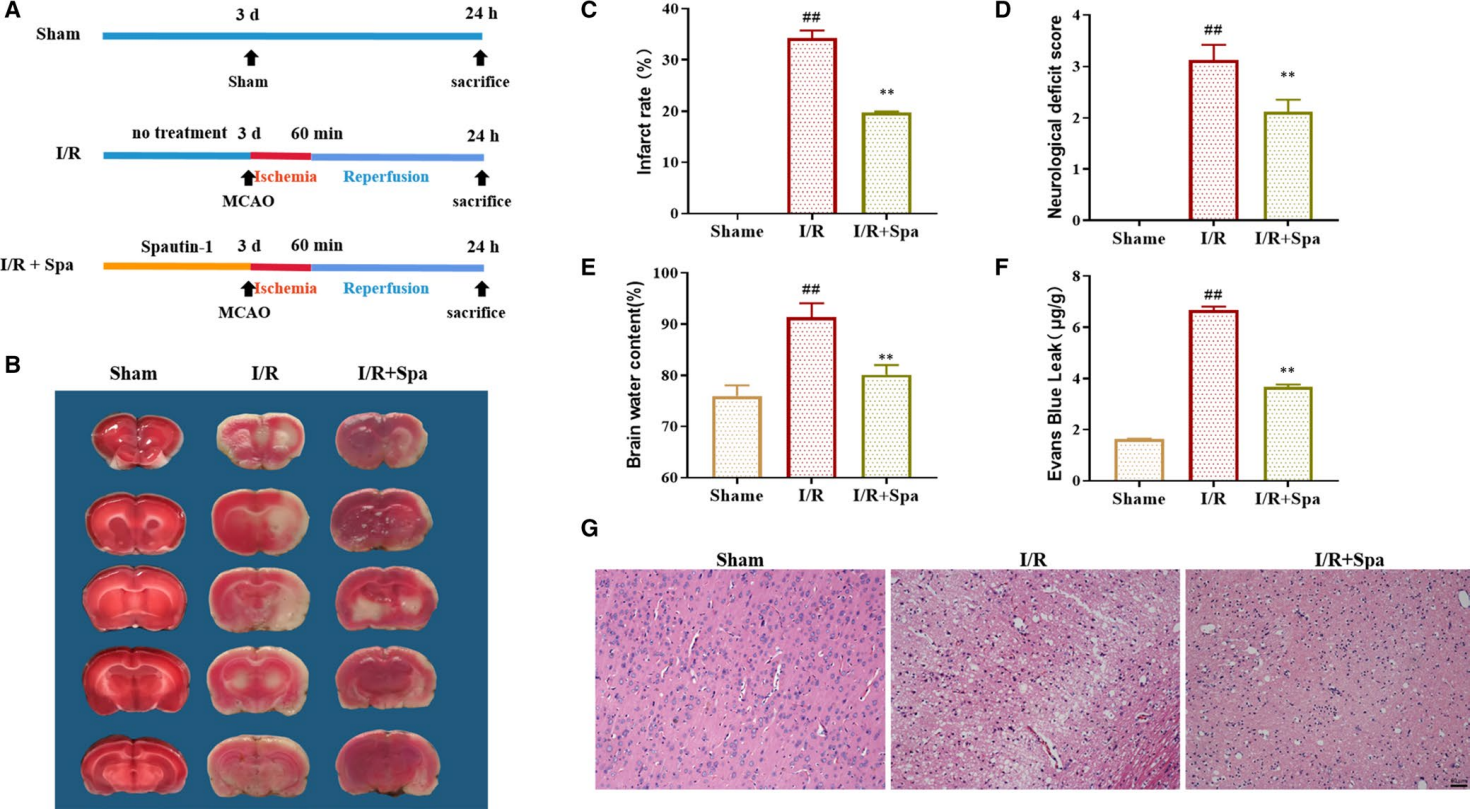
Spautin‐1 pre‐treatment ameliorates the injury of cerebral I/R. A, The procedure of ischaemia/reperfusion treatment in rats; B, Images of cerebral infarct size assessed by TTC staining. The red area represents the non‐ischaemic area, and the white area represents the corresponding ischaemic area. C, Histogram of infarct rate in brain tissues (n = 3); D, Neurological function assessed by Longa score (n = 8); E, Brain water content of rat in each group (n = 3); F, The leakage of Evans Blue in each group (n = 3); G, Pathological images of brain tissue assessed by H&E (n = 3). Sham, the sham operation group; I/R, the group with ischaemia/reperfusion group; I/R + Spa, the group with ischaemia/reperfusion plus Spautin‐1. Data are expressed as the mean ± SD, ^#^ *P* < .05 and *^##^P* < .01 compared with the Control group; *P < .05 and **P < .01 compared with the Model group

To further investigate the role of USP13, we generated an MCAO‐induced experimental ischaemia/reperfusion model in rats. Figure 4A showed the process of ischaemia/reperfusion in rats. TTC staining results showed that the infarct size of the I/R group was significantly larger than that of the sham operation group (*P* < .01), although Spautin‐1 treatment could significantly reduce the infarct size after MCAO surgery (*P* < .01, *P* < .01, Figure 4B,C). In addition, as shown by the neurological score, the score was significantly lower of the I/R group compared with the Sham group (*P* < .01), although Spautin‐1 treatment could significantly increase the score (*P* < .01, Figure 4D). Brain water content increased significantly after MACO surgery, and pre‐treatment with Spautin‐1 significantly reduced the water content (*P* < .01, Figure 4E). Ischaemia‐reperfusion injury to brain tissue could significantly increase the penetration of EB. However, Spautin‐1 reduced EB infiltration (*P* < .01, Figure 4F). As shown in Figure 4G, the brain tissue in the Sham group was normal; with no obvious vacuolar space, the cells were densely and evenly arranged; and the nucleoli were clearly visible. However, I/R caused severe damage to the brain tissue, many vacuoles appeared in the brain tissue of the I/R group, and the cells became sparse and disorderly. Compared with the I/R group, Spautin‐1 pre‐treatment observably alleviated the pathological abnormalities caused by I/R injury. The above results indicated that Spautin‐1 could reduce the area of cerebral infarction and improve neurological function in rats with cerebral ischaemia‐reperfusion.

### 3.5 Spautin‐1 pre‐treatment suppresses the expressions of autophagy and pyroptosis‐related proteins

**FIGURE 5:**
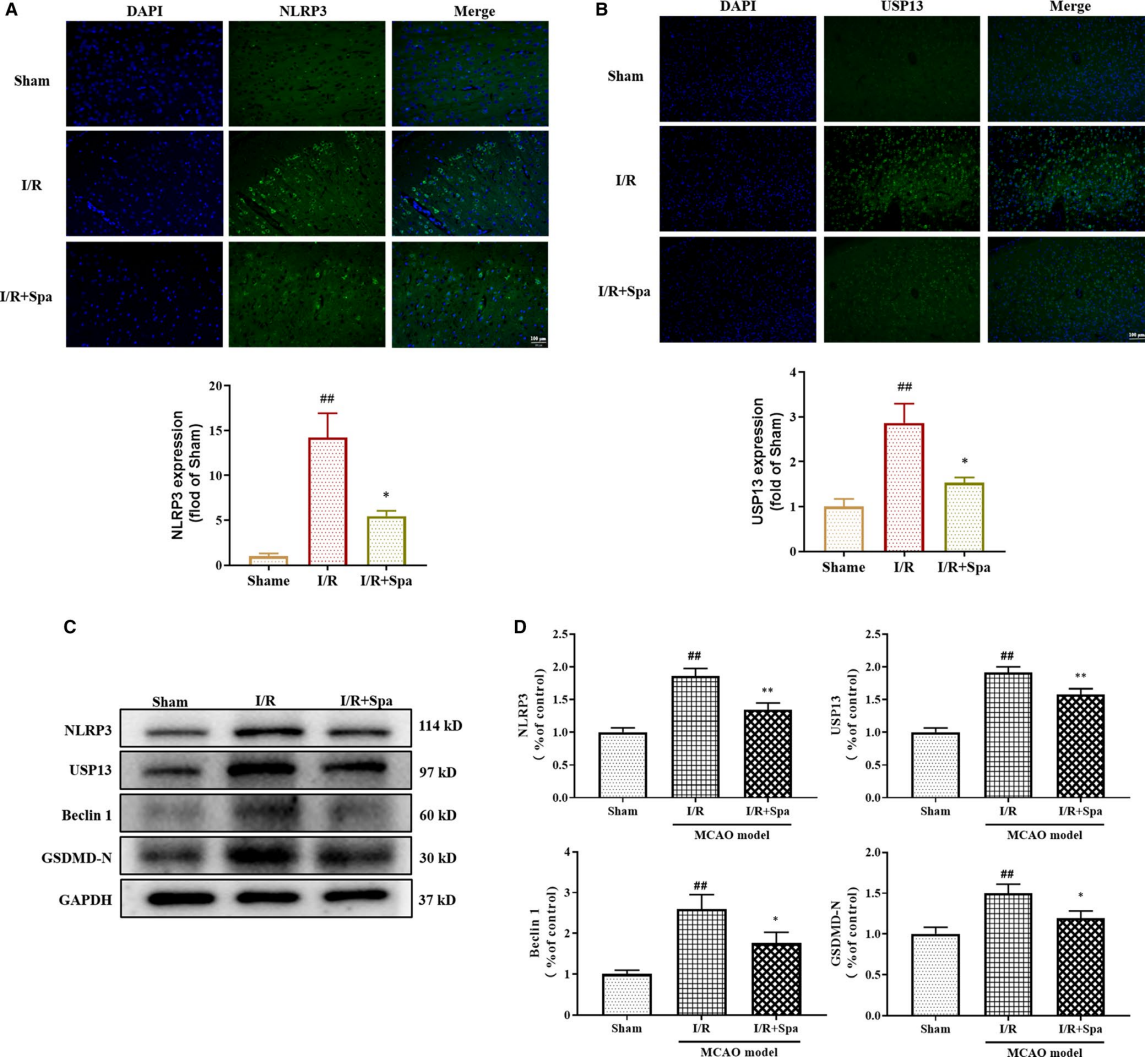
Effect of Spautin‐1 pre‐treatment on the autophagy and pyroptosis‐related proteins of brain tissue after CI/RI. A, The expression of NLRP3 in MCAO brain tissue by immunofluorescence (n = 3); B, The expression of USP13 in MCAO brain tissue by immunofluorescence (n = 3); C, Western blot analysis of the expression of autophagy and pyroptosis‐related proteins in brain; D, Densitometric analysis of autophagy and pyroptosis‐related proteins relative expression. Data are expressed as the mean ± SD, ^#^ *P* < .05 and *^##^P* < .01 compared with the Control group; *P < .05 and **P < .01 compared with the Model group

The expressions in rat brain tissues were determined by immunofluorescence of NLRP3 and USP13 (Figure 5A,B). The fluorescence intensity of NLRP3 and USP13 was significantly increased in the I/R group compared with the sham group, whereas the fluorescence intensity of NLRP3 and USP13 was significantly decreased in the I/R + Spa group relative to the sham group (*P* < .01). In order to detect the expressions of autophagy and focal death proteins in vivo, the effect of Spautin‐1 on the expressions of USP13, Beclin 1, NLRP3 and GSDMD‐N in the brain tissue of MCAO rats was examined by Western blot. The results showed that compared to the Sham group, the expressions of USP13, Beclin 1, NLRP3 and GSDMD‐N were significantly increased in the I/R group, and the expressions in the I/R + Spa group showed down‐regulated trends compared with the I/R group (Figure 5C,D).

## 4 DISCUSSION

This study investigated whether anti‐autophagy and anti‐inflammatory effects contribute to the protective effects of CI/RI, and further validated the possible mechanism of action of autophagy inhibition on protection against cerebral ischaemia‐reperfusion injury. The results confirmed the protective effect of autophagy inhibition in MCAO rats, and inhibition of autophagy significantly reduced the infarct size and improved the neurological score of rats. In vitro results indicated that autophagy inhibition increased cell viability and reduced the accumulation of intracellular ROS and expressions of pyroptosis‐related proteins. In addition, we determined that Spautin‐1 reduced autophagy and ferroptosis activation through inhibition of USP13, which may be a key mechanism by which autophagy inhibition protects the brain from cerebral ischaemia‐reperfusion injury.

Globally, cerebral ischaemia has high mortality and disability rate.^1^ In addition to oxidative stress and apoptosis, autophagy and inflammation accompanied by pyroptosis are considered as novel mechanisms of stroke pathology.^8^ In most cases, autophagy facilitates the stable maintenance of the intracellular environment by transferring substrates, such as necrotic organelles, to lysosomes. Studies have shown that autophagy activation is important for neuroprotection against cerebral ischaemia‐reperfusion injury.^24^, ^25^ However, excessive autophagy causes neuronal death to a large extent under cerebral ischaemia‐reperfusion conditions.^26^ The role of autophagy in cerebral ischaemia‐reperfusion needs to be further investigated.

Spautin‐1 was further used to investigate the mechanism of autophagy activation on cerebral ischaemia‐reperfusion injury, and Spautin‐1 is as an inhibitor of USP13. USP13 stabilizes the Vps34 complex to promote autophagy, and therefore, inhibition of USP13 reduces autophagy.^27^ In the present study, we found that elevated expression levels of Beclin‐1 and LC3II/I were accompanied by increased infarct size and decreased cell viability, suggesting that autophagy is disruptive in I/R rats and cellular models. Therefore, the hypothesis of this study is that USP13 down‐regulation protects the brain from ischaemia‐reperfusion injury by inhibiting autophagy. As expected, USP13 overexpression inhibited autophagy and survival of PC12 cells under OGD/R conditions, and Spautin‐1 treatment counteracted these effects. Based on these results, we hypothesized that down‐regulation of autophagic activation due to USP13 inhibition may have neuroprotective effects on cerebral ischaemia‐reperfusion injury. Consistent with our findings, studies show that autophagy inhibition has the neuroprotection effect against cerebral ischaemia in rats.^28^

Autophagy is associated with the activation of inflammatory microsomes NLRP3 under conditions of oxidative stress.^29^ We investigated whether inflammation is involved in this process, in addition to investigating whether inhibition of USP13 in a model of cerebral ischaemia‐reperfusion injury ameliorates the injury caused by autophagy. It reported that autophagy plays a key role in regulating mitochondrial integrity, ROS generation and subsequent NLRP3 inflammasome activation.^30^ Intracellular ROS content is also increasing with increased autophagy. NLRP3 inflammasomes consist of NLRP3, ASC and Caspase‐1. Previous studies have shown that NLRP3 activates Caspase‐1, cleaves GSDMD to form GSDMD‐N, which induces cell membrane perforation, cell rupture, and release of contents, causing ferroptosis. Therefore, high expression of Caspase‐1 and GSDMD is an indicator of pyroptosis.^31^, ^32^ The expressions of pyroptosis‐related proteins such as NLRP3, Caspase‐1 and GSDMD‐N were consistent with the trend of the autophagy marker LC3II/I, indicating that it was autophagy‐dependent.

In conclusion, we demonstrate that inhibition of autophagy can effectively mitigate cerebral ischaemia‐reperfusion injury and that this protective effect may be achieved through autophagy‐dependent pyroptosis. These data help to identify mechanisms by which autophagy inhibition exerts a protective role after cerebral ischaemia‐reperfusion and provide new ideas for protection against cerebral ischaemia‐reperfusion injury.

## CONFLICT OF INTEREST

The authors declare no conflict of interest.

## AUTHOR CONTRIBUTION

**Hui Liu:** Conceptualization (equal); Data curation (equal); Writing‐original draft (equal). **Zongbo Zhao:** Formal analysis (equal); Methodology (equal); Writing‐original draft (equal). **Tao Wu:** Data curation (equal); Methodology (equal). **Qiu Zhang:** Project administration (equal); Validation (equal); Visualization (equal). **Fenying Lu:** Software (equal); Validation (equal). **Jie Gu:** Project administration (equal); Software (equal). **Tingwang Jiang:** Resources (equal); Writing‐review & editing (equal). **Jianzhong Xue:** Resources (equal); Supervision (equal).

## Data Availability

Data will be available from the corresponding author upon reasonable request.
